# Genome‐wide CRISPR/Cas9 screening for therapeutic targets in NSCLC carrying wild‐type TP53 and receptor tyrosine kinase genes

**DOI:** 10.1002/ctm2.882

**Published:** 2022-06-12

**Authors:** Qianqian Wang, Jun Li, Jing Zhu, Jiaqi Mao, Chao Duan, Xiao Liang, Lingyun Zhu, Mengyan Zhu, Zhihong Zhang, Fan Lin, Renhua Guo

**Affiliations:** ^1^ Department of Oncology the First Affiliated Hospital of Nanjing Medical University Nanjing China; ^2^ Department of Oncology the Affiliated Jiangning Hospital of Nanjing Medical University Nanjing China; ^3^ Department of Cell Biology School of Basic Medical Sciences Institute for Brain Tumors & Key Laboratory of Rare Metabolic Diseases Nanjing Medical University Nanjing China; ^4^ Department of Bioinformatics Nanjing Medical University Nanjing China; ^5^ Department of Pathology the First Affiliated Hospital of Nanjing Medical University Nanjing China

**Keywords:** CRISPR/Cas9 screen, MDM2, NSCLC, RG7388

## Abstract

**Background:**

Targeted drugs have greatly improved the therapeutic outcome of non‐small cell lung cancer (NSCLC) patients compared with conventional chemotherapy, whereas about one‐third of patients are so far not suitable for targeted therapy due to lack of known driver oncogenes such as a mutated receptor tyrosine kinase (RTK) genes. In this study, we aimed to identify therapeutic targets for this subgroup of NSCLC patients.

**Methods:**

We performed genome‐wide CRISPR/Cas9 screens in two NSCLC cell lines carrying wild‐type TP53 and receptor tyrosine kinase (wtTP53‐RTK) genes using a GeCKO v2.0 lentiviral library (containing 123411 sgRNAs and targeting 19050 genes). MAGeCKFlute was used to analyse and identify candidate genes. Genetic perturbation and pharmacological inhibition were used to validate the result in vitro and in vivo.

**Results:**

The Genome‐wide CRISPR/Cas9 screening identified MDM2 as a potential therapeutic target for wtTP53‐RTK NSCLC. Genetic and pharmacological inhibition of MDM2 reduced cell proliferation and impaired tumour growth in the xenograft model, thus confirming the finding of the CRISPR/Cas9 screening. Moreover, treatment by a selective MDM2 inhibitor RG7388 triggered both cell cycle arrest and apoptosis in several NSCLC cell lines. Additionally, RG7388 and pemetrexed synergistically blocked the cell proliferation and growth of wtTP53‐RTK tumours but had limited effects for other genotypes.

**Conclusions:**

We identified MDM2 as an essential gene and a potential therapeutic target in wtTP53‐RTK NSCLC via a genome‐wide CRISPR/Cas9 screening. For this subgroup, treatment by RG7388 alone or by its combination with pemetrexed resulted in significant tumour inhibition.

## Graphical abstract

- Genome‐wide CRISPRCas9 screening identified MDM2 as a potential target for wtT53‐RTK NSCLC cells.
- MDM2 inhibitor RG7388 selectively kill wtT53‐RTK cells but is less effective against NSCLC cells carrying other genotypes.
- RG7388 and pemetrexed worked synergistically in blocking proliferation and tumor growth of wtT53‐RTK NSCLC.

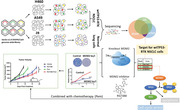

## 1 INTRODUCTION

Lung cancer is one of the most common malignant tumours affecting human health. The mortality rate of lung cancer ranks first and second in males and females, respectively.^1^ Non‐small cell lung cancer (NSCLC) is the most common histologic subtype, and it accounts for 80%–85% of all lung cancer cases.^2^ In the past decade, the extensive studies on oncogenic drivers of NSCLC revealed several essential mechanisms including angiogenesis, apoptosis, proteasome regulation, and cell cycle control involved in the tumourigenesis and development of NSCLC. These discoveries led to the development of a number of small molecule‐based targeted treatments that exhibited interesting anti‐tumour activities in preclinical and clinical studies. Among them, small molecular inhibitors targeting alterations in receptor tyrosine kinases (RTK) demonstrated excellent efficacy and low toxicity, and some of these have been approved to treat NSCLC patients.^3^, ^4^ Till today, targeted therapeutics have greatly reformed the treatment of NSCLC due to the better efficacy and lower incidence of adverse events. American Society of Clinical Oncology (ASCO), European Society of Oncology (ESMO), and National Comprehensive Cancer Network (NCCN) recommended targeted therapy as the first choice for patients with advanced NSCLC.^5^, ^6^, ^7^ However, the prerequisite for involving targeted drugs to achieve desired efficacy is the presence of aberrant alterations of several major oncogenes, such as EGFR and ALK. In 2016, Tsao et al. summarized the frequency of molecular aberrations that drive oncogenes in lung adenocarcinoma and the currently available targeted therapeutics against these oncogenic proteins.^3^ They reported the absence of any known oncogenic driver genes in 31% of lung adenocarcinoma cases.^3^ A recent study published in this year reported 21.7% Asian and 39.8% Western NSCLC patients carrying no actionable molecular alteration.^8^ Therefore, current targeted agents are not available for about one‐third of NSCLC patients.

TP53 gene is a well‐known tumour suppressor gene, participating in a series of biological processes such as cell differentiation, cell cycle regulation, apoptosis, and DNA repair.^9^ For NSCLC patients with mutant TP53 and wild‐type RTK, anti‐PD‐1/PD‐L1 is a good choice, because TP53‐mutated tumours were characterized by higher PD‐L1 expression and more potent immunogenicity, which means better response to checkpoint inhibitor.^10^, ^11^ However, for patients harbouring wild‐type TP53 and wild‐type RTK, named wtTP53‐RTK, neither immunotherapy nor targeted therapy can achieve good curative effects, which is unbearable in the individualized treatment today. Therefore, we aimed to identify new drug targets for this subgroup of patients to further improve the overall efficacy for NSCLC patients.

CRISPR/Cas9 gene knockout screen is a high‐throughput screening method that can systematically study gene functions and their role in cellular fitness genome‐wide based on CRISPR/Cas9 technology.^12,^, ^13^ CRISPR/Cas9 screen includes positive and negative selection screens. A positive selection screen refers to the identification of the sgRNAs enriched in cells after the screening, which can be used to study the mechanism of drug resistance genes for an example; on the contrary, a negative selection screen identifies sgRNAs markedly depleted in all cells after the screening, and provides a new method to study essential survival genes.^14^ Due to the successful application of CRISPR/Cas9 screen in the identification of new drug targets for various types of cancers,^12^, ^14^, ^15^, ^16^ we chose it to find genes that are essential for cell survival in NSCLC cell lines. As a result, MDM2 was identified as a new therapeutic target for the wtTP53‐RTK NSCLC. Furthermore, the anti‐tumour efficacy of MDM2 inhibitor RG7388 was also evaluated as a single‐agent therapy or in combination with the first‐line chemotherapeutic in different models.

## 2 MATERIALS AND METHODS

### 2.1 Cell lines and culture

Human NSCLC cell lines (NCI‐H460, A549, NCI‐H1299) and HEK293T were obtained from the Cell Bank of the Chinese Academy of Sciences (Shanghai, China). Human normal bronchial epithelial cell line BEAS‐2B was a gift from Dr. Y. Sun (Nanjing Medical University, Jiangsu, China). All the above cell lines were maintained in RPMI‐1640 (Gibco‐Invitrogen) or DMEM (Gibco‐Invitrogen, CA, USA) supplemented with 10% fetal bovine serum (VACCA, Shanghai, China) and penicillin (100 U/ml)–streptomycin (100 μg/ml) less than 15 passages, and authenticated by Biowing Biotech (Shanghai, China). These cell lines were cultured in a humid incubator (5% CO_2_, 37°C).

### 2.2 Lentivirus production

HEK293T cells were seeded into sixteen 15‐cm dishes (5 × 10^6^ cells/dish) for 24 h before transfection. When the cell density reaches 60%–70%, a mixture containing 14 μg of lentiCRISPR plasmid library (GeCKO v2.0 library; Addgene Plasmid #52961), 7 μg of pVSVg, and 10.5 μg of psPAX2 was transfected to each 15‐cm dish HEK293T cells. The cells were replenished with a fresh medium after 18 h post‐transfection. After another 72 h, the supernatant containing the lentivirus was collected and filtered through a 0.45‐μm low protein binding membrane. Subsequently, the virus was ultracentrifuged at 24000 rpm at 4°C for 2 h, and then resuspended in DMEM supplemented with 1% BSA at 4°C overnight. Stored at −80°C.

### 2.3 CRISPR/Cas9 screening with LentiCRISPRv2 library

Genome‐wide CRISPR knockout screening was performed as described.^13^ As the GeCKO v2.0 library contains 123 411 sgRNAs targeting 19050 genes, about 1.6×10^8^ NCI‐H460, A549, and BEAS‐2B cells need to be infected to achieve 300‐fold coverage at a multiplicity of infection of 0.3–0.5. Cells were seeded in 12‐well plates for 24 h and then infected with lentivirus by centrifuging at 2000 rpm for 2 h at 37°C in the presence of 8 μg/ml polybrene to increase transduction efficiency. After the spin, cells were replenished with fresh media (without polybrene) and cultured for another 24 h. Then, those cells were cultured in 15‐cm dishes to an appropriate density. Puromycin was then added into cells for 7 days at 1 μg/ml for A549 and BEAS‐2B, and 2 μg/ml for NCI‐H460 cells. 4 × 10^7^ cells were harvested and stored at −80°C for subsequent genomic DNA (gDNA) isolation (as Day 0). Meanwhile, other 4 × 10^7^ cells were subcultured every 3 days to maintain adequate sgRNA library complexity. On the 14th day, 4 × 10^7^ cells were collected for gDNA isolation and next‐generation sequencing (as Day 14).

### 2.4 Genomic DNA sequencing and data analysis

Genomic DNA was isolated using the Blood and Cell Culture DNA Maxi Kit (QIAGEN) according to the manufacturer's instructions, followed by two rounds of PCR procedure to identify and amplify sgRNAs as described previously.^14^ Primers sequences to amplify sgRNAs for the first PCR were:

1F: AATGGACTATCATATGCTTACCGTAACTTGAAAGTATTTCG
1R: CAGTTTACCCCGCGCCACCTTCTCTAGGCACCGGA

Primers for the second PCR were:

2F: AATGGACTATCATATGCTTACCGTAACTTGAAAGTATTTCG
2R: CCAACTTCTCGGGGACTGTGGGCGATGTGCGCTCTG

Amplified sgRNAs were purified using the QIAquick Gel Extraction Kit (Qiagen) and quantified, multiplexed, sequenced in Illumina Hiseq.

MAGeCKFlute is an algorithm specifically designed to analyse CRISPR screening data.^17^ By analysing the FASTQ file, a beta score was generated for each target gene to measure the degree of selection after the gene was perturbed. A negative beta score indicates negative selection by profiling the depletion of sgRNAs targeting essential survival genes.

### 2.5 Stable cell line generation

The lentiCRISPR v2.0 plasmid (Addgene Plasmid #52961) containing MDM2 sgRNA was transfected into HEK293T cells to produce lentivirus. The sequence:

sg1: CACCGAGACACTTATACTATGAAAG;
sg2: CACCGTATATTATGACTAAACGAT;

A549, NCI‐H460 and NCI‐H1299 cells were infected with lentivirus and selected by puromycin to generate stable sgMDM2 cells. The KO status was validated by Western blot analysis.

### 2.6 Cell viability assays

Cell viability was monitored using the Alamar Blue assay, which indicates cellular metabolic activity. Cells were plated in a 96‐well plate and exposed to different conditions of treatment. At the end of exposure, add 110 μL of 10% Alamar Blue (Yeasen, Shanghai, China) per well in the medium and incubated for 4 h at 37°C and 5% CO_2_ in the dark. Fluorescence was read at an excitation wavelength of 534 nm and an emission wavelength of 584 nm (Fluoroskan Ascent FL analyser). For each set of conditions, perform three times in triplicate. All the data points were normalized to the values of mock‐treated samples.

### 2.7 Clonogenic survival assay

Cells were seeded at a density of 500 cells/ml in a 2‐ml culture medium in six‐well plates, and cells were allowed to attach for about 12 h to ensure single cells were attached to the bottom of each well at the time of drug‐exposure. After addition of the drug, the plates were incubated for 14 days at 37°C with 5% CO_2_ in a humidified incubator. After incubation, the culture medium was discarded, and cells were washed by PBS. Finally, the cells were fixed with 4% paraformaldehyde and stained with a Giemsa Stain solution. Finally, all colonies were manually scored.

### 2.8 Cell cycle analysis and apoptosis assay

For analysis of the cell cycle, cells were seeded in six‐well plates and incubated with different treatments. Cells were washed twice in ice‐cold PBS and fixed in 75% cold ethanol overnight at 4°C. After removing the ethanol, the fixed cells were washed with pre‐cold PBS twice, treated with 0.1 mg/ml RNase A at 37°C in the dark for 30 min, and stained with 50 μg/ml of propidium iodide (PI) (Sigma‐Aldrich, St Louis, USA) solution in the dark for 5 min. Finally, the stained cells were analysed with a FACS Calibur flow cytometer.

For apoptosis assay, cells in the logarithmic growth phase were seeded in six‐well plates; then were incubated at 37°C and 5% CO_2_ condition. After incubation with different treatments, the cells were rinsed with pre‐cold PBS twice, and then stained with Annexin V and PI according to the manufacturer's instructions. A flow cytometer was used to analyse the stained cells within 1 h.

### 2.9 Western blotting

Proteins were extracted by RIPA lysis buffer (Beyotime Biotechnology, Shanghai, China) containing 0.1 M PMSF and protease inhibitors (Roche, Basel, Switzerland). Proteins were quantified by the BCA Protein Assay Kit (Yeasen, Shanghai, China). The supernatant was collected after centrifugation, which was stored at −80°C. Proteins were fractionated by SDS‐PAGE, and then transferred to PVDF membranes (Merck Millipore, MA, USA), and blocked with 3% bovine serum albumin for 2 h at room temperature. The membranes were probed with primary antibodies at 4°C overnight. The following primary antibodies were used for immunoblotting: MDM2 (ProteinTech, #19058‐1‐AP), p21(Santa‐Cruze, sc817), p53 (#2524), Cleaved‐caspase3 (#9661), Bim (#2933), Bak (#6947), Bcl‐xl (#2764), Survivin (#2808) (all from CellSignaling, Danvers, USA), GAPDH (#AC002) (ABclonal, Hubei, China). For the apoptosis antibody array, an Apoptosis Array Kit (CST, #ARY009) containing antibodies specific for 37 apoptosis‐related proteins was used. Then, the membranes were washed by TBST (1×) and incubated with a corresponding horseradish peroxidase‐conjugated secondary antibody for 2 h at room temperature. The immunoreactive bands were visualized using an ECL kit (Beyotime, Shanghai, China) and analysed using Imager software (Tanon, Shanghai, China).

### 2.10 Animal studies

Mice were housed and handled according to institutional guidelines complying with local legislation. All experiments with animals were approved by the animal experiment committee of the Nanjing Medical University. NOD/ShiLtJGpt‐*Prkdc^em26Cd52^Il2rg^em26Cd22^*/Gpt (NCG, male or female, 5–8 weeks, 18–22 g) and BALB/C mice were purchased from Jiangsu GemPharmtech co., Ltd (Nanjing, China) and were adapted to the environment for a week before the experiment. NCG mice were injected subcutaneously with 0.2 ml cell suspension containing 5×10^6^ live NCI‐H460 wt or *MDM2* KO cells to induce tumour xenografts. The tumour size was measured with a vernier caliper every 3 days, and tumour volumes were calculated using the following formula: length × width^2^/2. For treatment, mice were randomly divided into groups with similar mean tumour volumes of 50 to 150 mm^3^. RG7388 was purchased from Selleck (Selleck Chemical, USA) and was administered by oral gavage at a dose of 50 mg/kg q.d. Pemetrexed was obtained from Selleck and was administered intraperitoneally at a dose of 100 mg/kg b.i.w. (twice per week). Once the tumour volume reached 2000 mm^3^, mice were sacrificed by cervical dislocation.

### 2.11 Statistical analysis

All in vitro experiments except the screens and the Western Blotting were at least repeated three times. The data of in vitro experiments were expressed as mean ± standard deviation (SD). The data of tumour growth are presented as mean ± standard error of the mean (SEM). Data analysis was performed by Graphpad 8.0 software (GraphPad Software Inc., La Jolla, USA). The *p*‐values were calculated using a two‐tailed Student's *t*‐test, and *p* < 0.05 was considered to indicate statistical significance. The following *p* values considered significant: *p < 0.05; **p < 0.01; ***p < 0.001; ****p < 0.0001.

## 3 RESULTS

### 3.1 Genome‐wide CRISPR/Cas9 screens identified MDM2 as a new drug target for NSCLC

**FIGURE 1:**
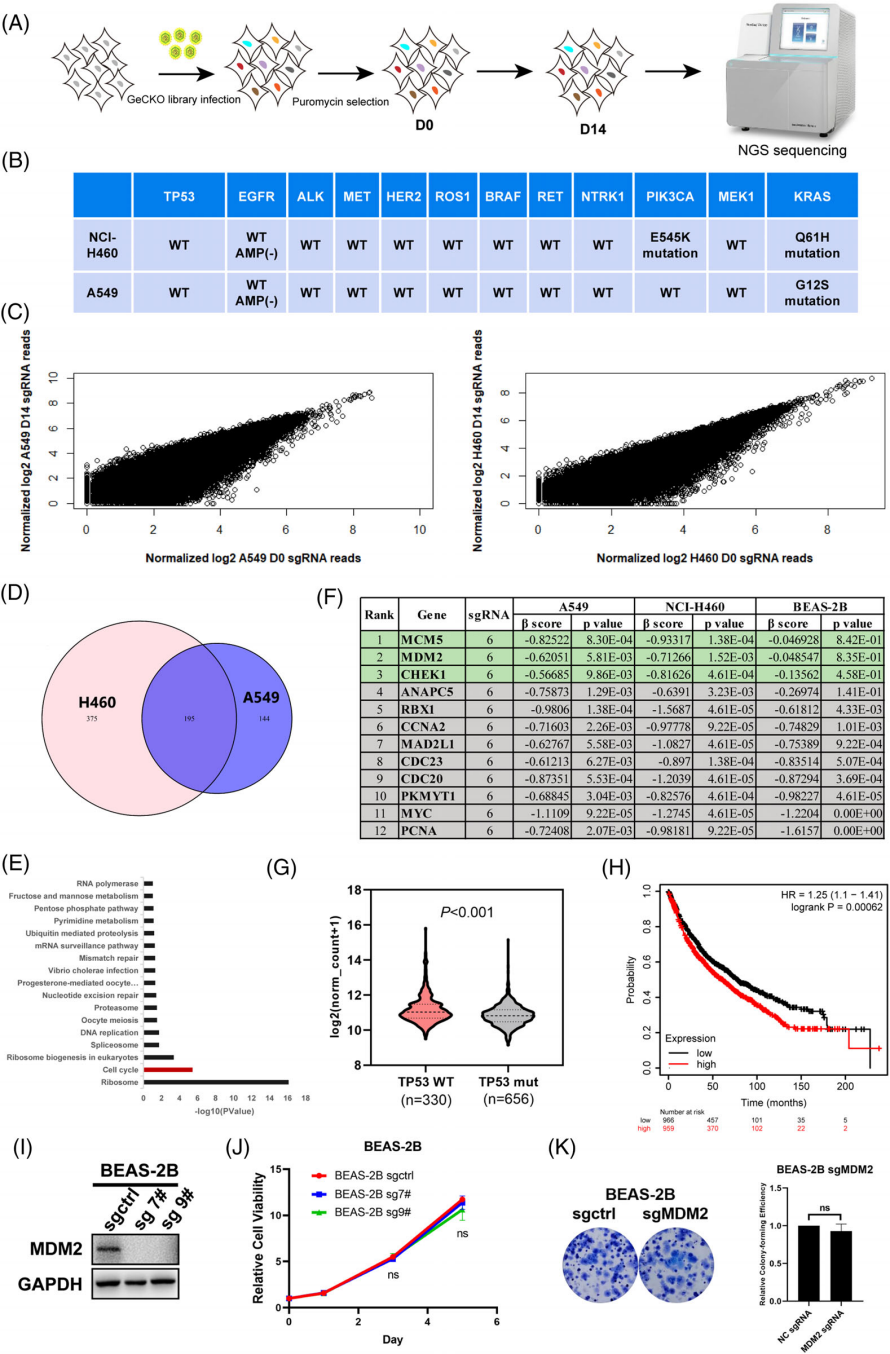
Genome‐wide CRISPR/Cas9 screens identified MDM2 as a potential therapeutic target for NSCLC. (A) Schematic diagram of the CRISPR/Cas9 screens. (B) The mutated status of lung cancer driver oncogenes in A549 and NCI‐H460 cell lines. (C) The scatterplot of sgRNAs reads count in A549 and NCI‐H460 cells. (D) Venn diagram depicting the NSCLC potential targets defined as the intersection of screening in A549 and NCI‐H460. (E) The KEGG enrichment analysis on the intersected targets of screening in A549 and NCI‐H460. (F) The list of potential targets based on cell cycle pathway. The top three candidates whose β score > −0.2 were highlighted in green. (G) The expression of MDM2 in TP53 wild‐type and TP53 mutant NSCLC tumour tissues. (H) Kaplan–Meier plots for MDM2 (217373_x_at) expression‐based overall survival analysis of lung adenocarcinoma. (I) Expression levels of MDM2 of sgctrl and *MDM2* KO BEAS‐2B cells (lung epithelial cell line). (J) Alamar Blue assays were performed to determine the cell viability of sgctrl and *MDM2* KO clones of BEAS‐2B cells. (K) Colony formation assays of sgctrl and *MDM2* KO BEAS‐2B cells. Representative images (left) and quantification (right) are shown (*n* = 3)

We performed two genome‐wide CRISPR/Cas9 screens in both NCI‐H460 and A549 cell lines which harbour wild‐type TP53 and wild‐type RTK genes (Figure 1A). At the same time, we also performed a genome‐wide CRISPR/Cas9 screen in BEAS‐2B cells, a normal bronchial epithelial cell line, as control. The status of some common driver gene mutations of NCI‐H460 and A549 is listed in Figure 1B. In this study, we used GeCKO v2.0 lentiviral library containing 123411 sgRNA, targeting 19050 genes for potential drug targets screening. The abundance of sgRNA in NCI‐H460 and A549 cells was compared between D0 and D14 (Figure 1C). We used MAGeCK‐Flute Integrative Analysis Pipeline to identify the depleted sgRNAs (negative selection, β score < −0.2 as threshold) and the intersection of NCI‐H460 and A549, and finally obtained 195 candidate genes^17^ (Figure 1D). KEGG pathway enrichment analysis of these genes revealed that the depleted genes were primarily enriched in the cell cycle pathway (Figure 1E). In this pathway, 12 genes were identified and ranked by the reverse order of the β score in absolute value of BEAS‐2B cells. β scores and *p*‐values in NCI‐H460, A549, and BEAS‐2B cell lines were also listed in Figure 1F, and the top three candidates of the cell cycle pathway were highlighted in green. Among them, we chose the E3 Ubiquitin Protein Ligase *MDM2*, a well‐known oncogene as a potential therapeutic target for wtTP53‐RTK NSCLC for further study. Bioinformatic analysis based on TCGA data indicated that *MDM2* was highly expressed in TP53 wild‐type compared with TP53 mutant NSCLC (including lung adenocarcinoma and lung squamous cell carcinoma), suggesting overexpression of *MDM2* could be beneficial for the growth of TP53 wild‐type cancer (Figure 1G). Moreover, Kaplan–Meier analysis of the lung carcinoma data in the TCGA database using an online survival analysis web server (http://kmplot.com/analysis/) revealed that high *MDM2* expression was associated with poorer overall survival in patients (Figure 1H). Importantly, knockout *MDM2* in a normal human bronchial epithelial cell line BEAS‐2B cells (Figure 1I) did not affect its proliferation (Figure 1J) and colony formation (Figure 1K), suggesting MDM2 is not essential for survival and proliferation of normal lung cells. Overall, the above results suggested that MDM2 is a potential therapeutic target for NSCLC, so we focused on *MDM2* in the following study.

### 3.2 Knockout MDM2 led to reduction of cell proliferation and tumour growth

**FIGURE 2:**
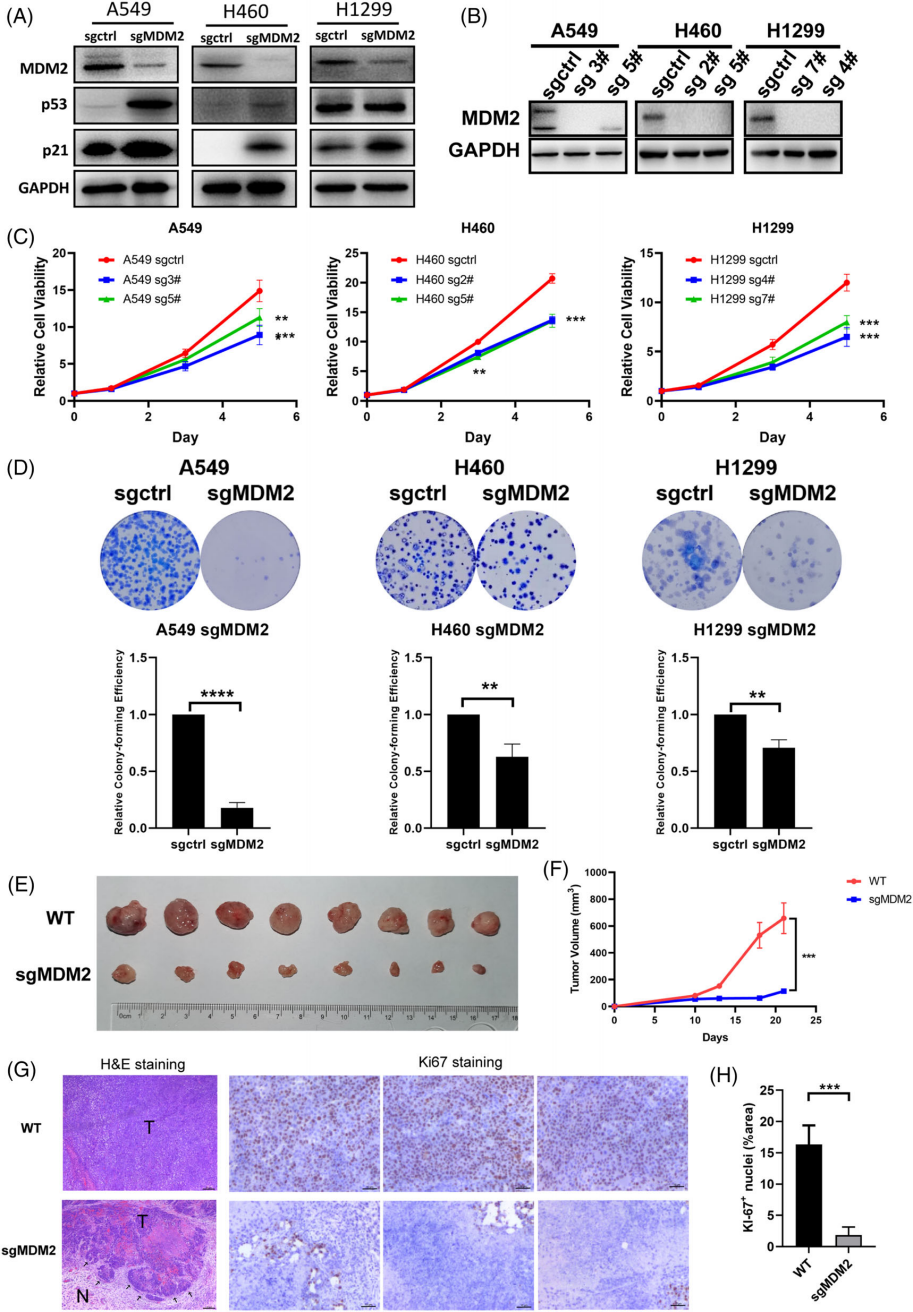
Knockout MDM2 led to reduction of cell proliferation and tumour growth. (A) Expression levels of the p53 pathway‐related proteins of sgctrl and *MDM2* KO A549, NCI‐H460, and NCI‐H1299 cells. (B) Expression levels of MDM2 of sgctrl and *MDM2* KO A549, NCI‐H460, and NCI‐H1299 clones. (C) Alamar Blue assays were performed to determine cell viability of sgctrl and *MDM2* KO A549, NCI‐H460, and NCI‐H1299 clones. (D) Colony formation assays of sgctrl and MDM2 KO A549, NCI‐H460, and NCI‐H1299 cells. Representative images (upper) and quantification (down) are shown (*n* = 3). (E,F) Images and tumour volume curve of WT and MDM2 KO NCI‐H460 cells‐derived xenograft tumors. (G) H&E and Ki67 IHC staining of xenograft tumours. Scale bar = 50 μm. (H) Quantitative of Ki67 nuclei staining area. Error bars represent SEMs. *, *p* < 0. 1; **, *p* < 0.01; ***, *p* < 0.001; ****, *p* < 0.0001

To validate the screening results, *MDM2* stable knockout cell lines were generated. We knocked out the *MDM2* gene in A549, H460, and H1299 cell lines using CRISPR/Cas9 technology and validated the deficiency of MDM2 in the cell lysates by western blotting. Upon deletion of *MDM2*, both the p53 and p21 levels in A549 and NCI‐H460 were elevated as expected, given that the MDM2‐mediated p53 ubiquitination and degradation were abolished. Although the TP53 is deleted in NCI‐H1299 cell line, a weak band of p53 protein in H1299 cells which is presumably a truncated variant of p53 could still be detected. Interestingly, we found that the p21 level was still elevated, indicating that knockout MDM2 can cause upregulation of p21 via other pathway instead of the classic p53 route (Figure 2A). Next, two individual clones from each knockout cell lines were isolated and were subjected to Western blotting analysis. As Figure 2B shown, the MDM2 were completely deleted in each of the single‐cell derived clones. Cell viability assay showed that both *MDM2*‐knockout (KO) clones significantly impaired the cell viability of A549, NCI‐H460, and NCI‐H1299 cells relative to the control (knockout with a non‐specific control sgRNA) cells (Figure 2C). Consistently, the colony formation assessing the long‐term effect of MDM2 knockout showed a marked reduction of the colony number in KO cells relative to control cells (Figure 2D).

To further validate that MDM2 is a candidate target for wtTP53‐RTK NSCLC, we implanted NCI‐H460 MDM2 WT (control) and KO cells in NCG immunodeficient to evaluate their tumourigenicity in the xenograft model. As a result, MDM2 KO xenografts were substantially smaller than those with WT MDM2, and the tumour growth curve of the mice bearing KO xenografts showed a remarkable retarded growth compared with mice in the control group (Figure 2E,F). Hematoxylin‐eosin (H&E) staining of the fixed tumour tissue showed the less aggressive and invasive growth of the MDM2 KO cells compared with the WT cells; as shown in Figure 2G, the front edge of the MDM2 KO tumour was clearly visible. In contrast, the growth of MDM2 WT tumour appeared to be more aggressive since a clear tumour edge was hard to be found. In Figure 2G,H, the Ki‐67 nuclei staining revealed that the knockout MDM2 resulted into dramatic reduction of the Ki‐67 positive proliferative cells compared with those in the WT xenografts.

### 3.3 RG7388 inhibited cell proliferation via induction of apoptosis and cell cycle blockage and suppressed the wtTP53‐RTK NSCLC tumour growth in vivo

**FIGURE 3:**
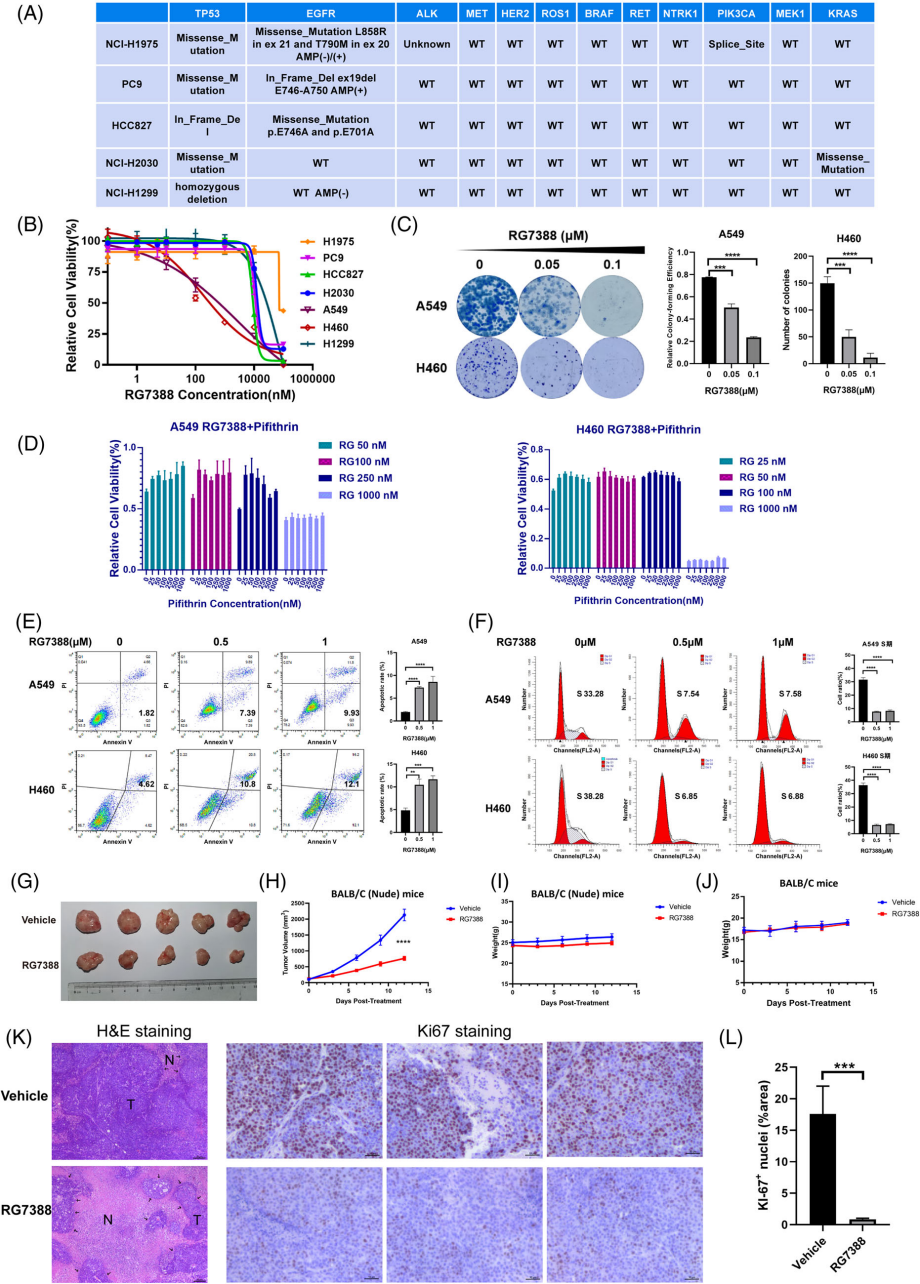
RG7388 inhibited cell proliferation via induction of apoptosis and cell cycle blockage and suppressed the wtTP53‐RTK NSCLC tumour growth in vivo. (A) The mutated status of lung cancer driver oncogenes in mutTP53 NSCLC cell lines. (B) Cell viability of multiple NSCLC cell lines after treatment for 72 h with different doses of MDM2 inhibitor RG7388. (C) Colony formation assays of A549 and NCI‐H460 cells with different doses of RG7388. Representative images (left) and quantification (right) are shown (*n* = 3). (D) Cell viability of A549 and NCI‐H460 cells after treatment for 72 h with different doses of MDM2 inhibitor RG7388 and p53 inhibitor Pifithrin. (E) Flow cytometric analysis detects the apoptotic of A549 and NCI‐H460 cells with different doses of RG7388 treatment for 72 h. Representative images (left) and quantification (right) are shown (*n* = 3). (F) Flow cytometric analysis determines the cell‐cycle distribution of A549 and NCI‐H460 cells with different doses of RG7388 treatment for 72 h. Representative images (left) and quantification of S‐phase (right) are shown (*n* = 3). (G,H) Images and tumour volume curve of NCI‐H460 xenograft tumours after vehicle or 50 mg/kg RG7388 treated. (I) Bodyweight curve of NCI‐H460 xenograft mice after vehicle or RG7388 treated. (J) Bodyweight curve of normal BALB/C mice after vehicle or RG7388 treated. (K) H&E and Ki67 IHC staining of xenograft tumours after vehicle or RG7388 treated. Scale bar = 50 μm. (L) Quantitative of Ki67 nuclei staining area. Error bars represent SEMs. *, *p* < 0. 1;**, *p* < 0.01; ***, *p* < 0.001; ****, *p* < 0.0001

To further corroborate the results from gene perturbation, we examined the anti‐tumour effect of pharmacological inhibition of MDM2 using a selective small molecular inhibitor, RG7388. First, we included both mutant TP53 and/or mutant RTK NSCLC cell lines together with wtTP53‐RTK cell lines for anti‐proliferation assessment. The genotypes of these cell lines are listed in Figures 1B and 3A. As shown in Figure 3B, RG7388 inhibited the proliferation of wtTP53 cell lines in a dose‐dependent manner, with an average IC50 below 2 μM. In comparison, it hardly had any inhibitory effect against cells carrying both TP53 and/or RTK mutations below 10 μM. Similarly, RG7388 effectively suppressed the colony formation of wtTP53‐RTK cell lines A549 and NCI‐H460 at 0.05 and 0.1 μM (Figure 3C). Another evidence to support that MDM2 targeted treatment is uniquely effective in wtTP53 NSCLC cells is shown in Figure 3D, as inhibition of the wild‐type TP53 by pifithrin‐α (a selective TP53 inhibitor) in A549 and NCI‐H460 cells counteracted the anti‐proliferative activity of RG7388. The interactions between RG7388 and pifithrin‐α appeared to be antagonistic as most of the high single agent (HSA) values between the two drugs in dose matrices were negative (Figure S1A,B) and a number of CI values > 1 (Figure S1C,D).

To investigate whether the anti‐tumour effect of RG7388 was due to cell death or growth arrest, we analysed the apoptosis and cell cycle blockage induced by RG7388. Flow cytometry analyses of A549 and H460 cells demonstrated that treatment of 0.5 μM RG7388 significantly increased the Annexin V‐positive fraction and 1 μM further aggravated this early apoptotic fraction of cells (Figure 3E). Flow cytometric analysis of cell cycle in PI‐stained A549 and H460 cells treated with or without RG7388 showed that RG7388 significantly reduced the proportion of cells at S‐phase at 0.5 μM but caused no further reduction at 1 μM, suggesting 0.5 μM RG7388 is sufficient to block the cell cycle (Figure 3F). Taken together, these results showed that MDM2 inhibitor RG7388 exerts an anti‐tumour effect via induction of both apoptosis and cell cycle arrest and these findings are in line with the dual role of P53 partaking in both apoptosis and G1‐phase checkpoint pathway.

To evaluate the therapeutic effect of MDM2 inhibitor in vivo, RG7388 or vehicle solution was administered orally to mice carrying subcutaneous xenograft formed by NCI‐H460 parental cells. After oral gavage of vehicle or 50 mg/kg RG7388 q.d. for a total period of 12 days, the tumour volumes in the RG7388 treatment group were significantly smaller than those in the control group (Figure 3G) and the tumour growth of the treatment group was markedly retarded relative to the vehicle‐treated group (Figure 3H). During the treatment, the body weights of all mice were monitored and there was no statistical difference found between the treated and control groups, indicating that the RG7388 is well tolerated by mice (Figure 3I). Moreover, treatment of RG7388 with the same dose of 50 mg/kg also did not cause significant loss of body weight in immunocompetent BALB/C mice (Figure 3J). The H&E staining of the fixed tumour tissues showed that the necrotic areas in the tumour tissue in the RG7388 treatment group were generally bigger than the control group (Figure 3K). The Ki67 staining showed the Ki67 positive proliferative cells were markedly reduced by the RG7388 treatment in comparison with the vehicle treated control group (Figure 3K,L). Therefore, we concluded that the pharmacological inhibition of MDM2 by a small molecular inhibitor RG7388 in vivo also led to significant suppression of wtTP53‐RTK tumours.

### 3.4 RG7388 and pemetrexed combined treatment exerted synergistic inhibition against wtTP53‐RTK NSCLC cells

**FIGURE 4:**
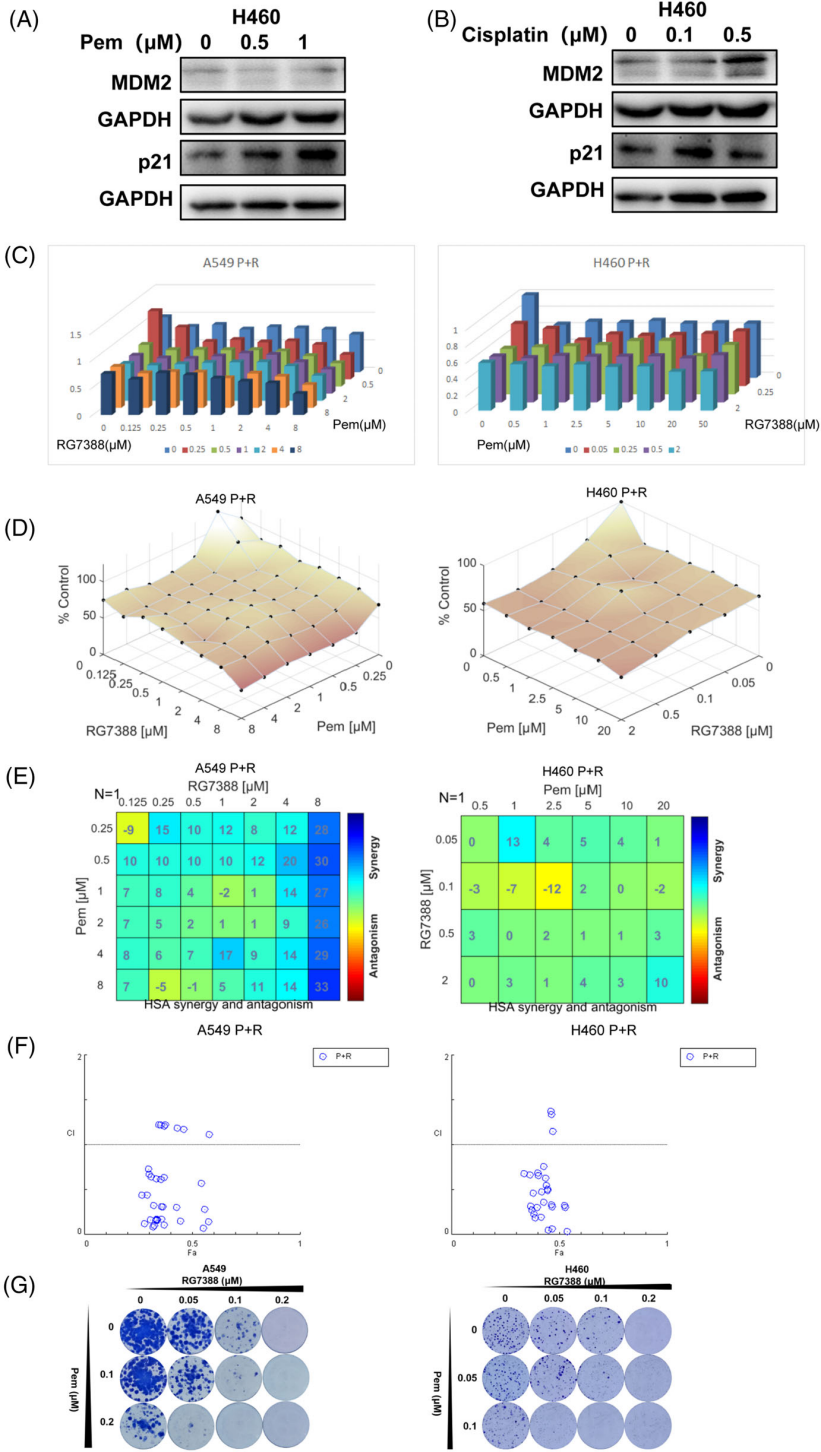
RG7388 and pemetrexed combined treatment exerted synergistic inhibition against wtTP53‐RTK NSCLC cells. (A) NCI‐H460 cells were immunoblotted to assess the expression levels of the p53 pathway‐related proteins after being treated with different doses of pemetrexed for 24 h. (B) Western blot analysis of expression level of p53 pathway‐related proteins in NCI‐H460 cells treated with different doses of cisplatin for 24 h. (C,D) Dose‐response histograms and surface plots of RG7388 and pemetrexed single and combinatorial titration treatment A549 and NCI‐H460 cells for 72 h. (E) Synergy plots generated by Combenefit showing analysis of the interaction between RG7388 and pemetrexed resulted in HSA values. HSA values > 0 indicate synergistic effects. (F) Fa‐CI plots of analysis of RG7388 and pemetrexed interaction in A549 and NCI‐H460 cell lines. CI values < 1.0 means synergistic effects. (G) Colony formation assays of A549 and NCI‐H460 cells with different doses of RG7388 and pemetrexed single and combinatorial titration treatment

To improve the efficacy of RG7388, we further explored the effect of RG7388 in combination with chemotherapy drugs. Pemetrexed and cisplatin are two current standard chemotherapies for lung adenocarcinoma. Pemetrexed but not cisplatin could increase the expression of the downstream protein p21 of the p53‐MDM2 pathway, so pemetrexed was chosen to combine with RG7388 (Figure 4A,B).

To determine the combined effect of RG7388 and pemetrexed, A549 and NCI‐H460 cells were treated with a different dose titration matrix of the two drugs. The outcome of single or combined treatment of the two drugs was present in dose‐response 3D bar charts and surface plots to demonstrate their combined effects (Figure 4C,D). Next, we used Bliss and HSA synergy analytic models to determine whether the interactions between RG7388 and pemetrexed are synergistic, additive, or antagonistic.^18^ Our results showed that most of the HSA values were above 0, suggesting the interaction between RG7388 and pemetrexed was primary synergistic (Figure 4E). In addition, Fa‐CI plots (fraction affected Combination Index) revealed that most combinations had a CI value below 1.0, indicating a general synergistic instead of additive interaction between the two drugs (Figure 4F). In the colony formation experiments that required long‐term treatment, the combination of RG7388 and pemetrexed also exhibited synergistic suppression against colony formation in three NSCLC cell lines (Figure 4G). In comparison, RG7388 and pemetrexed combined treatment only resulted in very limited inhibition in mutTP53‐mutRTK PC9 cells and HCC827 cells (Figure S2A,B), and failed to induce synergistic proliferation‐inhibitory effects as indicated by a majority of negative HSA values of drug–drug combinations at different concentrations (Figure S2C,D). Although quite some CI values were below 1.0, all Fa values < 0.5 suggest a lack of robust activity against PC9 and HCC827 cells by the combined treatment (Figure S2E,F). It is still unknown whether the EGFR mutation or other mechanism was involved in its poor responsiveness to the RG7388 or pemetrexed‐RG7388 combined treatment. Altogether, these data demonstrated a synergistic inhibition effect of RG7388 and pemetrexed to wtTP53‐RTK NSCLC cells.

### 3.5 Combined treatment of RG7388 and pemetrexed synergistically induced cell apoptosis

**FIGURE 5:**
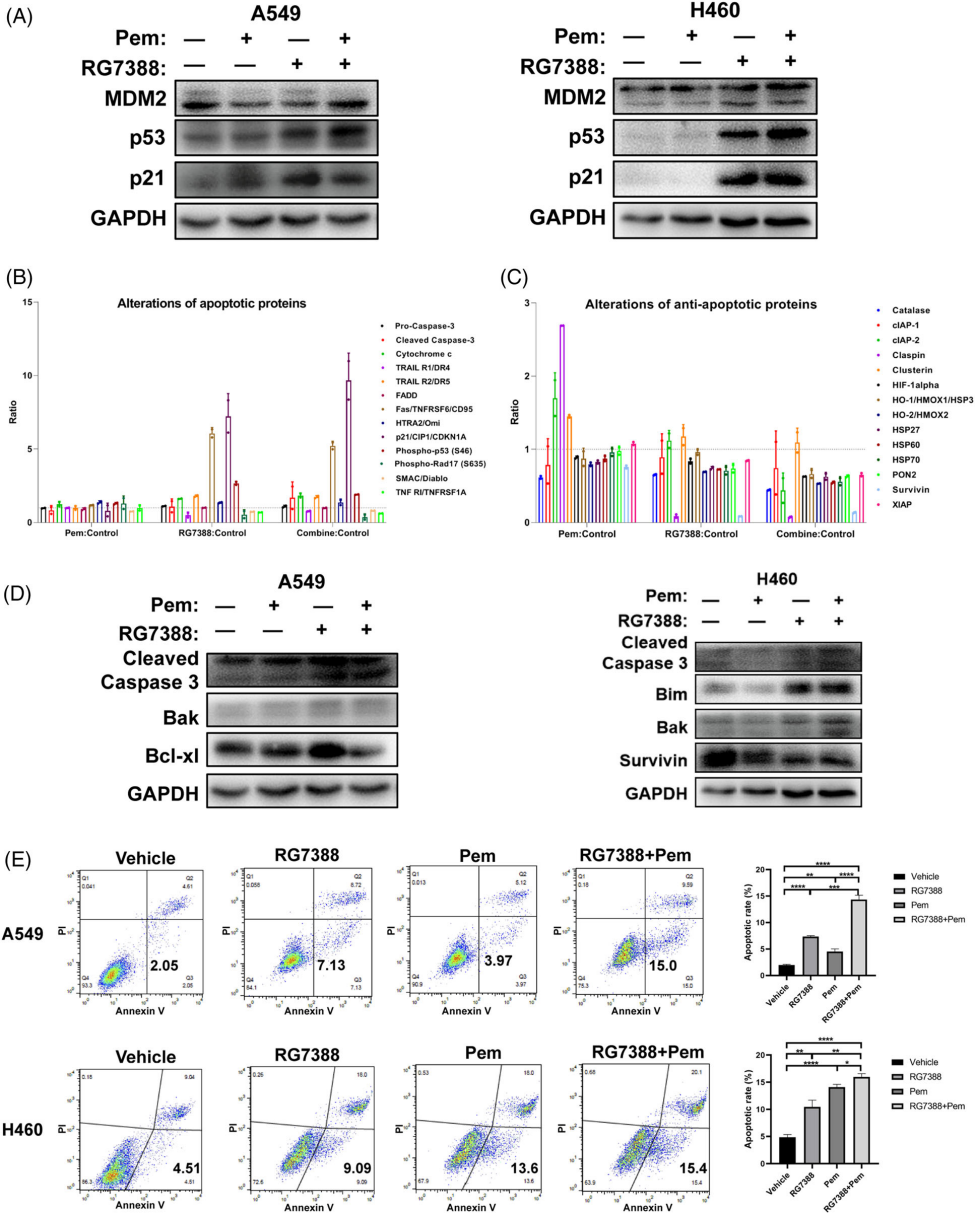
Combined treatment of RG7388 and pemetrexed synergistically induced cell apoptosis. (A) The p53 pathway proteins expression level changes were detected by Western blots in A549 and NCI‐H460 cells after RG7388 and pemetrexed single or combinatorial treated for 24 h. (B,C) Analysis of alterations in the apoptotic (B) and anti‐apoptotic proteins (C) in A549 cells treated with RG7388 and pemetrexed alone or in combination using an apoptosis antibody array. (D) The expression of apoptosis‐related proteins was detected by Western blots in A549 and NCI‐H460 cells after RG7388 and pemetrexed single or combinatorial treatment for 24 h. (E) Flow cytometric analysis detects the apoptosis of A549 and NCI‐H460 cells with different doses of RG7388 and pemetrexed single or combinatorial treatment for 72 h. Representative images (left) and quantification (right) are shown (*n* = 3). Error bars represent SEMs. *, *p* < 0. 1;**, *p* < 0.01; ***, *p* < 0.001; ****, *p* < 0.0001

In all wtTP53‐RTK cell lines, RG7388 treatment successfully increased the expressions of p53, and the addition of pemetrexed further aggravated the trends (Figure 5A). At the same time, for mutTP53‐mutRTK PC9 cells, neither pemetrexed nor RG7388 treatment was capable of increasing the expression of p53 or p21 due to lack of wild‐type p53 protein, and the level of p21 was even reduced when the two drugs were combined (Figure S2G). Although the TP53 contains an in frame deletion in HCC827 cells, we did observe elevated levels of p53 and p21 by RG7388 treatment, so the p53 was probably still functioning (Figure S2H). We also detected the expression of p53 and MDM2 in both mut‐TP53 and/or mut‐RTK cell lines (Figure S2I). As TP53 triggers cellular apoptosis, we speculated that the addition of pemetrexed also aggravated the apoptosis induction by RG7388 treatment. 37 apoptosis‐associated proteins were analysed by an Apoptosis Antibody Array Kit to determine the differentially expressed proteins in A549 cells treated with RG7388 and pemetrexed alone or in combination. In comparison with the untreated control, the expression of a number of apoptotic proteins showed an upward trend after the combined treatment of RG7388 and pemetrexed, while the levels of survivin, claspin, and other anti‐apoptotic proteins decreased to varying degrees. In comparison with RG7388 treatment, the combined treatment further increased the levels of cleaved‐Caspase 3 and p21, and decreased catalase, cIAP2, and a few other anti‐apoptotic proteins (Figure 5B,C). Furthermore, western blotting demonstrated that the expression of cleaved‐Caspase 3 was increased upon combined treatment of RG7388 and pemetrexed relative to single‐agent treatment (Figure 5D). Last, we performed flow cytometric analyses to examine whether the addition of pemetrexed to RG7388 further enhances its induction of apoptosis. Indeed, the results demonstrated that the combined treatment significantly increased the annexin V‐positive fraction compared with those treated by RG7388 alone (Figure 5E). In summary, the above results confirmed that the combination treatment of RG7388 and pemetrexed further enhanced the apoptosis and anti‐tumour effect of RG7388 and pemetrexed‐based current standard treatment for lung adenocarcinoma with wtTP53‐RTK genotype.

### 3.6 Combined treatment of RG7388 and pemetrexed suppressed wtTP53‐RTK NSCLC tumour growth in vivo

**FIGURE 6:**
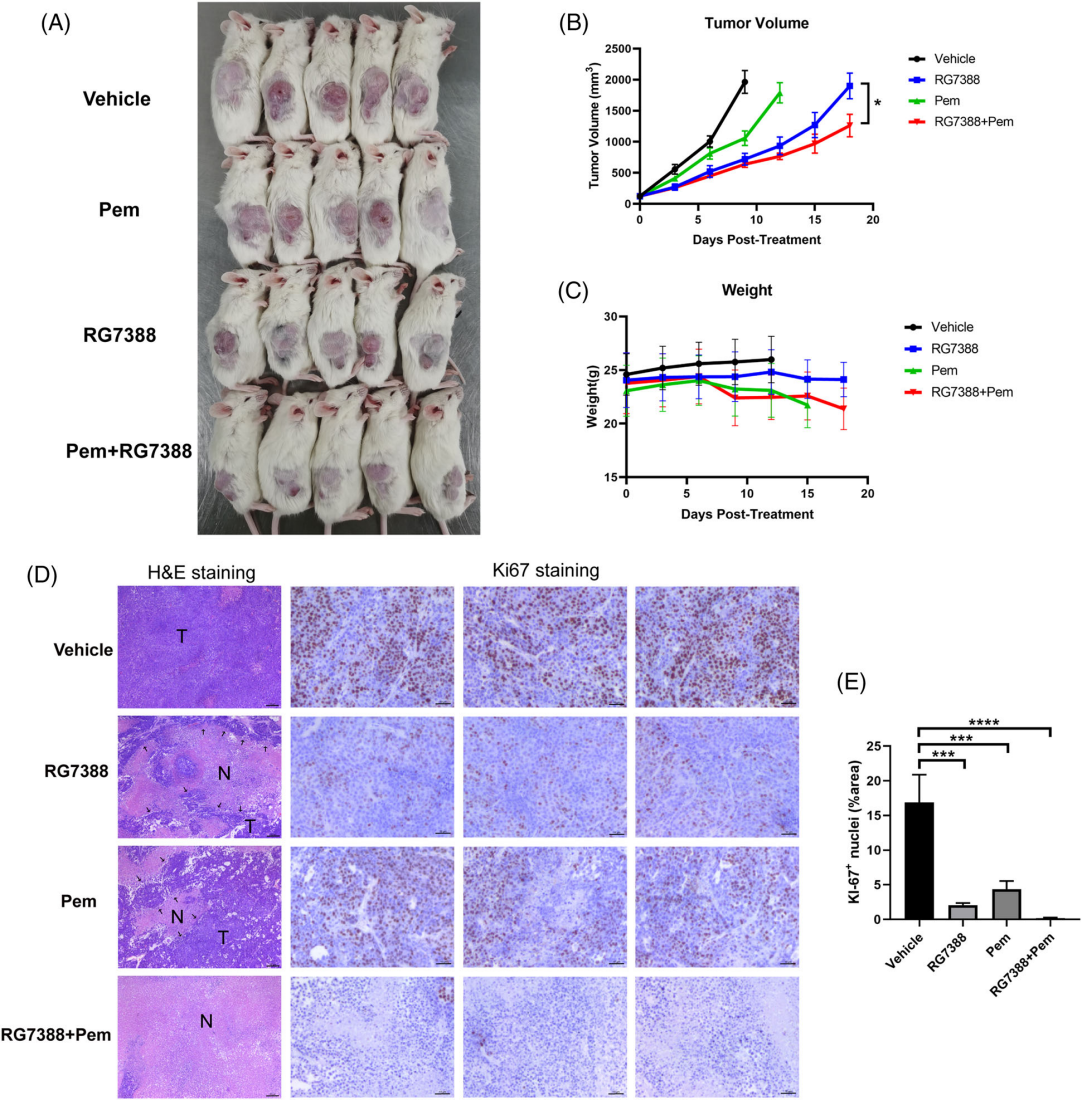
Combined treatment of RG7388 and pemetrexed suppressed wtTP53‐RTK NSCLC tumour growth in vivo. (A,B) Images and tumour volume curve of NCI‐H460 xenograft tumours after RG7388 (50 mg/kg q.d.) and pemetrexed (100 mg/kg b.i.w.) single or combinatorial treatment. (C) Bodyweight curve of NCI‐H460 xenograft mice after RG7388 and pemetrexed single or combinatorial treatment. (D) H&E and Ki67 IHC staining of xenograft tumours after RG7388 and pemetrexed single or combinatorial treatment. Scale bar = 50 μm. (E) Quantitative of Ki67 nuclei staining area. Error bars represent SEMs. *, *p* < 0. 1;**, *p* < 0.01; ***, *p* < 0.001; ****, *p* < 0.0001

To evaluate the efficacy of the combination treatment of RG7388 and pemetrexed in vivo, NCG mice bearing H460 xenografts were treated with RG7388 and/or pemetrexed by oral gavage and/or 100 mg/kg pemetrexed by i.p. injection b.i.w. As shown in Figure 6A, the volumes of subcutaneous tumours in the single‐agent RG7388 treated group were smaller than those in the control group but were bigger than those in the RG7388 and pemetrexed combined treatment group (Figure 6A). The tumour growth curve demonstrated that RG7388 treatment effectively restrained the H460 tumour growth, and the addition of pemetrexed further aggravated such an effect (Figure 6B). Interestingly, the tumour growth rate in the RG7388 group was slower than that of the pemetrexed group, suggesting that the anti‐tumour activity of RG7388 was superior to pemetrexed in treating wtTP53‐RTK NSCLC (Figure 6B). In addition, the body weight curves among all treatment groups were similar, suggesting that the toxicities of RG7388 and the combination treatment were well tolerated (Figure 6C). H&E staining showed that the combination of RG7388 prevented the aggressive growth of H460 tumour graft and caused dramatic tumour tissue necrosis (Figure 6D). Finally, Ki67 staining revealed that both pemetrexed and RG7388 treatment effectively eliminated the Ki67 positive proliferative tumour cells, whereas the combination of the two drug exhibit an even more potent in vivo anti‐proliferation effect (Figure 6D,E).

## 4 DISCUSSION

The primary goal of this study is to identify a novel therapeutic target for NSCLC patients who lack actionable molecular alteration which accounts for about 1/3 of the (worldwide) NSCLC population. This subgroup of patients carry neither EGFR mutation nor other RTK mutations, so it is somehow like the triple‐negative breast cancers, currently lacking effective therapy. Therefore, we performed a genome‐wide CRISPR/Cas9 knockout screen in wtTP53‐RTK NSCLC and normal lung cell lines to identify potential therapeutic targets for this subtype. Although the two screened NSCLC cell lines H460 and A549 have mutations in other genes such as KRAS and PIK3CA, the target we wanted to find should be uniformly effective for the genetic subgroup of wtTP53‐RTK NSCLC regardless of the statuses of other genes. After bioinformatics analysis, 12 candidate targets, including MDM2 were selected out. MDM2 was finally chosen for further evaluation not only because it ranked second in the cell cycle pathway but also because several small molecular inhibitors selectively targeting MDM2 are available to study the in vitro and in vivo efficacy against wtTP53‐RTK NSCLC.

*MDM2* is a known oncogene that negatively regulates p53 by its E3 ubiquitin ligase function. Amplification of its genes and/or overexpression of its protein further enhances tumourigenesis, according to previous studies.^19^, ^20^ Overexpression of MDM2 was frequently observed in various types of cancers, such as osteosarcoma, rhabdomyosarcoma, and fibrosarcoma,^21^, ^22^ and it was usually associated with poor prognosis. For NSCLC patients, several studies indicated that overall survival was significantly shorter in patients with *MDM2* amplification.^23^, ^24^, ^25^ In our study, bioinformatics analysis found that MDM2 was up‐regulated in lung adenocarcinoma and lung squamous cell carcinoma cells compared with normal tissue,^23^, ^26^ and the elevated level of MDM2 was associated markedly with short survival in NSCLC patients. As a primary negative regulator of p53, MDM2 controls its transcriptional activity, protein stability, and nuclear localisation.^9^, ^27^ First, as an E3 ubiquitin ligase, MDM2 mediates the ubiquitination and degradation of p53.^9^, ^28^ Moreover, MDM2 also binds to the active transcriptional region of p53 to inhibit the transcriptional promoting function of p53.^9^ Meanwhile, *MDM2* is also a target protein at the downstream of TP53. TP53 binds to the *MDM2* promoter and regulates the expression of MDM2 at the transcriptional level.^29^ Thus, an autoregulatory feedback loop was formed between p53 and MDM2, and MDM2 was able to affect the cell cycle progression and apoptosis by regulating p53 in the loop.^20^, ^30^, ^31^

Restoration of p53 activity by inhibition of the p53‐MDM2 interaction has been explored as a novel approach for cancer treatment, and a variety of inhibitors have been developed.^32^ RG7388 (idasanutlin) is a second‐generation clinical MDM2 inhibitor, inhibiting the interaction of p53‐MDM2.^32^ It effectively activated the p53‐MDM2 pathway, induced cell cycle arrest and apoptosis, and suppressed tumour growth, proving its potent inhibition against p53‐MDM2 interaction.^32^

Currently, the clinical investigation of the anti‐cancer efficacy of RG7388 is undergoing in several solid and haematological tumours. Despite the fact that RG7388 exhibited a good curative effect in the treatment of acute myeloid leukaemia, the phase 3 trial MIRROS combining idasanutlin and cytarabine was stopped in 2020 because it did not meet its primary goal of being superior to placebo plus cytarabine.^33^, ^34^ Therefore, the molecular basis and a genetic subgroup which can be benefited from MDM2 targeting therapy should be investigated in depth. In this study, RG7388 exhibited excellent therapeutic effect against the wtTP53‐RTK NSCLC, suggesting the genetic status of TP53 and RTK are crucial for the efficacy of MDM2 inhibitor. Although the anti‐cancer activity of RG7388 has been confirmed in a number of preclinical studies, whether it is an effective therapeutic for NSCLC is still unclear. We assessed the apoptosis induction and cell cycle blockage by RG7388 treatment, and the results showed that RG7388 had a significant effect on regulating apoptosis and cell cycle progression which was consistent with previous studies.^35^, ^36^ Moreover, the in vivo efficacy and tolerability for several types of cancers of RG7388 were also exhibited in previous studies.^35^, ^37^ It is noteworthy that the anti‐cancer activity of MDM2‐inhibitor is mainly dependent on a functional p53, and we further confined the potential beneficial group from MDM2 inhibition to NSCLC patients carrying wild‐type TP53 and RTKs to maximize the efficacy. So far, whether the wild‐type RTKs contribute to the sensitivity of RG7388 treatment as well as its underlying mechanism are uncertain, but by our reckoning, the p53‐mediated apoptosis may be attenuated by the activated mutation of EGFR or other RTKs. Comparing with cells harbouring sole KRAS or PI3K mutation, EGFR mutation can act on multiple signalling pathways in concert to well protect tumour cells from apoptosis. Other than that, EGFR and other RTKs activate JAK‐STAT3 and other pathways to confer the tumour cells resistance to apoptosis. In contrast, the cells with wild‐type TP53 and RTKs may be more vulnerable to MDM2 inhibition because the anti‐apoptosis signalling was relatively weak compared with those harbouring mutated TP53 and RTK genes. Obviously, more solid work is required to offer a clear answer for it.

To further improve the effectiveness of MDM2 inhibition, we combined RG7388 with pemetrexed, a first‐line chemotherapeutic agent for NSCLC and investigated their interaction and feasibility for clinical application. Pemetrexed is an inhibitor of the folate metabolism pathway, which inhibits the thymidylate synthase (TS, TYMS), the key enzyme of folate metabolism. As pemetrexed also exerts its anti‐cancer effect via p53 mediated apoptosis, a combined treatment of two drugs putatively aggravates the RG7388‐induced apoptosis. Indeed, we revealed that the interactions between pemetrexed and RG7388 were synergistic and the addition of pemetrexed further enhanced the RG7388‐induced apoptosis in NSCLC cells, but the detailed mechanism needs further exploration.^35^, ^38^

In conclusion, we successfully identified MDM2 as a potential therapeutic target for wtTP53‐RTK NSCLC via a genome‐wide CRISPR/Cas9 screen and confirmed the therapeutic potential using a small molecular inhibitor RG7388, both as a single agent or in combination with chemotherapeutic drugs. These findings provide a novel and feasible therapeutic solution for NSCLC patients carrying wild‐type TP53 and RTKs, who are not suitable for current targeted therapies.

## CONFLICT OF INTEREST

The authors declare no potential conflicts of interest.

## Supporting information

Supporting Information

## Data Availability

The data generated in this study are available upon request from the corresponding author.
