# Supplementary material for: Genome‐wide CRISPR/Cas9 screening for therapeutic targets in NSCLC carrying wild‐type TP53 and receptor tyrosine kinase genes
Source: Clin Transl Med. 2022 Jun 12;12(6):e882. doi: 10.1002/ctm2.882 (PMC9189421; doi:10.1002/ctm2.882)
Supplement: Supplementary file 1 — Supporting Information [file CTM2-12-e882-s001.docx]

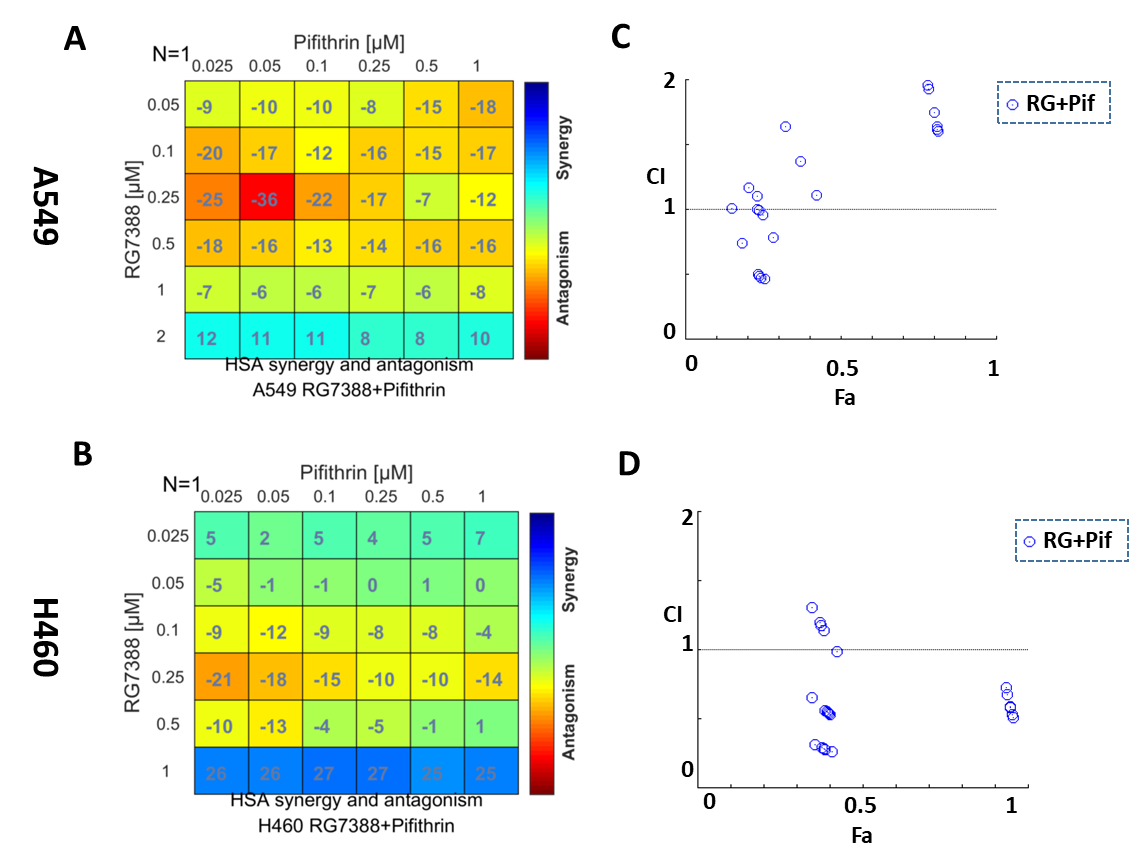


**Fig. S1. Combined treatment of RG7388 and Pifithrin α in wtTP53-RTK NSCLC cells. A and B**, Synergy plots generated by Combenefit showing analysis of the interaction between RG7388 and Pifithrin α and were quantified as HSA values in A549 and NCI-H460 cells. HSA values > 0 indicate synergistic effects. **C and D**, Fa-CI plots of analysis of RG7388 and Pifithrin α interaction in A549 and NCI-H460 cells. CI value < 1.0 means synergistic effects.


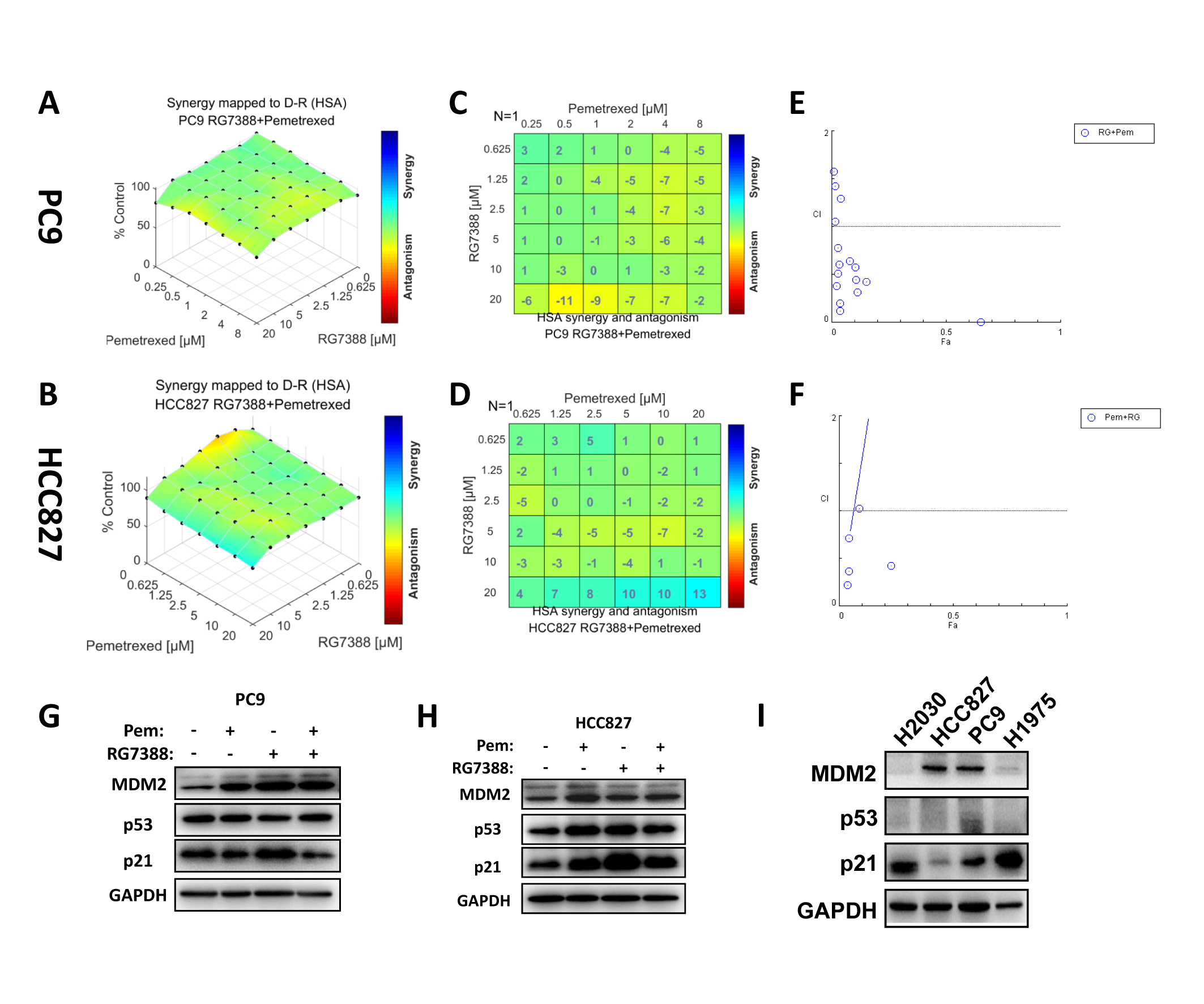


**Fig. S2. Combined treatment of RG7388 and pemetrexed in non-wtTP53-RTK NSCLC cells.**

**A and B**, Dose-response surface plots of RG7388 and pemetrexed single and combinatorial titration treatment NSCLC cells for 72 h in mutTP53 cell line PC9 and mutRTK cell line HCC827. **C and D**, Synergy plots generated by Combenefit showing analysis of the interaction between RG7388 and pemetrexed and were quantified as HSA values. HSA values > 0 indicate synergistic effects. **E and F**, Fa-CI plots of analysis of RG7388 and pemetrexed interactions. CI value < 1.0 means synergistic effects. **G and H,** The p53 pathway proteins expression level changes were detected by Western blots in PC9 and HCC827 cells after RG7388 and pemetrexed single or combinatorial treated for 24 h. **I,** The baseline expression of p53 pathway proteins of H2030, HCC827, PC9 and H1975 cell lines.
